# Bright’s Disease

**Published:** 1884-03

**Authors:** 


// HALL'S

### JOURNAL OF HEALTH

AND

MISCELLANY.

MONTHLY.

E. H. GIBBS, A. M., M. D., EDITOR.

FOUNDED IN 1854.

### BRIGHT'S DISEASE.

THERE is a prevalent opinion that this disease is far more common than formerly; but I question whether this is so. Indeed, the disease is more rare than is generally supposed. Until the discoveries of Doctor Bright, of London, the various affections of the kidneys were not so well defined, and any serious derangement of those organs was known simply as "kidney disease;" and the actual disease was seldom determined until it had passed beyond the reach of remedies. Now that the pathology of the disease is known there is a tendency to go to the extreme of confounding all affections that present one or more of its symptoms with Bright's disease. This *scare* is so vigorously worked by venders of quack medicines that one may meet a score of persons any day who sincerely believe that their lives have been saved by these *marvelous* remedies. Sometimes a person will observe that the urine is highly colored and on cooling a sediment settles in the bottom of the vessel. If he does not happen to realize that this is nature's method of relieving the system of materials that should not be retained, and, if otherwise well, that the best thing to do is to take no notice of it, he will probably rush to the nearest drug store and purchase for $1 a bottle of stuff, a few doses of which will generally restore the urine to an amber color, without sediment, but he might save 99 cents if he knew that as much common baking soda as could be dipped up with a ten-cent piece, dissolved in half a tumbler of cold water, and drank, would do the same thing more certainly and more safely. This very simple remedy should not be carried too far. Use it three or four times in 24 hours perhaps, and then don't repeat it for several days, or it will lose its effect, and might do injury to the digestive organs.

Whatever produces irritation of the kidneys disposes them to disease. When the body is suddenly chilled, so that perspiration is checked, the secretions are thrown back on the kidneys, whereby their labor is greatly increased. Liquor drinking is a prominent cause of kidney complaint. Rich and stimulating foods, particularly those known as nitrogenous foods, force a great deal of extra labor on the kidneys, and when such a cause is long continued is liable to result unfortunately.

It is safe to say that while nineteen-twentieths of supposed kidney diseases exist in the imagination, a majority of the cases of actual disease are due to excesses in eating and drinking and lack of sufficient exercise. Where there is a suspicion of disease of any kind go at once to a physician. It will save time, money, fretting and vain regrets, and perhaps life itself.

